# Genome-Wide Analysis of Oxidosqualene Cyclase Genes in *Artemisia annua*: Evolution, Expression, and Potential Roles in Triterpenoid Biosynthesis

**DOI:** 10.3390/cimb47070545

**Published:** 2025-07-14

**Authors:** Changfeng Guo, Si Xu, Xiaoyun Guo

**Affiliations:** 1 Guangxi Botanical Garden of Medicinal Plants, Nanning 530012, China; cfguo2020@163.com; 2 School of Life Science and Bioengineering, Jining University, Qufu 273155, China; xusi@jnxy.edu.cn

**Keywords:** *Artemisia annua*, oxidosqualene cyclase (OSC), triterpenoids, gene family, genome-wide analysis

## Abstract

Plant triterpenoids are structurally diverse specialized metabolites with significant ecological, medicinal, and agricultural importance. Oxidosqualene cyclases (OSCs) catalyze the crucial cyclization step in triterpenoid biosynthesis, generating the fundamental carbon skeletons that determine their structural diversity and biological functions. Genome-wide identification of OSC genes was performed using bioinformatics tools, including HMMER and BLASTP, followed by phylogenetic analysis, gene structure analysis, conserved domain and motifs identification, cis-regulatory element prediction, protein–protein interaction analysis, and expression profiling using publicly available transcriptome data from UV-B treated *A. annua* six-week-old seedlings. We identified 24 AaOSC genes, classified into CAS, LAS, LUS, and unknown subfamilies. Phylogenetic analysis revealed evolutionary relationships with OSCs from other plant species. Gene structure analysis showed variations in exon–intron organization. Promoter analysis identified cis-regulatory elements related to light responsiveness, plant growth and development, hormone signaling, and stress response. Expression profiling revealed differential expression patterns of AaOSC genes under UV-B irradiation. This genome-wide characterization provides insights into the evolution and functional diversification of the OSC gene family in *A. annua*. The identified AaOSC genes and their regulatory elements lay the foundation for future studies aimed at manipulating triterpenoid biosynthesis for medicinal and biotechnological applications, particularly focusing on enhancing stress tolerance and artemisinin production.

## 1. Introduction

Oxidosqualene cyclase (OSC) constitutes a family of enzymes that play pivotal roles in the biosynthesis of triterpenoids and sterols in plants. These enzymes catalyze the cyclization of the linear precursor 2,3-oxidosqualene into a diverse array of triterpene skeletons, which serve as the building blocks for various bioactive compounds. The importance of OSCs in plant metabolism is underscored by their involvement in the production of compounds with significant pharmacological activities, such as anti-inflammatory, anticancer, and antimicrobial properties [1,2,3,4].

*Artemisia annua* L., commonly known as sweet wormwood, is a medicinal plant renowned for its ability to produce artemisinin, a potent antimalarial sesquiterpenoid lactone [5,6,7]. In recent years, there has been a growing interest in understanding the biosynthetic pathways and regulatory mechanisms underlying artemisinin production in *A. annua* [8,9,10]. While significant progress has been made in elucidating the enzymes involved in artemisinin biosynthesis, the role of OSCs in this process remains largely unexplored.

Critically, OSC-mediated triterpenoid diversification extends far beyond species-specific metabolites like artemisinin, representing a universal adaptive strategy in flowering plants. Structurally diverse triterpenoids, such as saponins and lupeol derivatives, contribute significantly to plant defense by physically disrupting pathogen membranes and deterring herbivores [11,12]. Additionally, their antioxidant properties play a crucial role in mitigating oxidative damage caused by environmental stressors like UV-B exposure and water deficit [13,14]. This functional significance is underpinned by genomic analyses across angiosperms, which reveal that OSC gene family expansion and neofunctionalization strongly correlate with environmental specialization [15,16]. A compelling example is *Glycyrrhiza uralensis*, which evolved distinct β-amyrin oxidases enabling the production of the root-specific sweetener glycyrrhizin [17]. Collectively, these findings illustrate that OSC genes function as evolutionary toolkits for metabolic innovation driven by selective pressures.

Triterpenoids are abundant in *A. annua* and contribute significantly to the plant’s defense mechanisms and adaptation to environmental stress [18]. Despite their importance, the genome-wide identification, evolution, and expression patterns of OSC genes in *A. annua* have not been systematically studied. This knowledge gap limits our understanding of the triterpenoid biosynthetic pathways in this plant and hinders efforts to enhance the production of valuable triterpenoids through genetic engineering.

To date, more than 170 OSC genes have been biochemically characterized in plants, resulting in the identification of over 100 triterpene skeletons and a total of 61 distinct products [1,19]. The first β-amyrin synthase and cycloartenol synthase were isolated from *Pisum sativum* [20]. The cDNA of *Arabidopsis thaliana* was successfully transformed into yeast cells, leading to the identification of the first cycloartenol synthase gene in plants [21]. To date, a total of 13 OSC genes have been characterized in *Arabidopsis* [22,23]. The gene encoding β-amyrin synthase was isolated from ginseng roots through homologous cloning [24]. *PvOSC6* encoded β-amyrin synthase from *Prunella vulgaris*, while PvOSC2 functioned as a multifunctional synthase [25]. β-amyrin synthase genes have been identified in a variety of dicotyledonous plants [26,27,28,29], constituting the largest class of triterpenoid synthase genes discovered in plants to date. Moreover, β-amyrin synthase genes have also been cloned from monocotyledonous species [30,31]. Another significant OSC in plants is lupane synthase, which catalyzes the conversion of 2,3-oxidosqualene into lupeol, a pentacyclic triterpenoid. This enzyme was first isolated from *Olea europaea* and *Taraxacum officinale* [32]. Since then, lupane synthase genes have been identified and cloned from various plant species, including *Betula platyphylla*, *Glycyrrhiza glabra*, *Lotus japonicus*, *Bruguiera gymnorrhiza* and *Prunella vulgaris* [25,29,33,34]. Additionally, a multifunctional lupeol synthase, primarily producing lupeol with minor by-products, has been isolated from *Arabidopsis thaliana* and *Ricinus communis* [23,35,36].

James et al. successfully engineered triterpene production in *Saccharomyces cerevisiae* by utilizing a β-amyrin synthase derived from *A. annua* [37]. The predicted protein showed a high degree of similarity to other β-amyrin synthases, sharing up to 86% amino acid sequence identity. Expression of this gene in *S. cerevisiae*, followed by GC/MS analysis, confirmed its function as a β-amyrin synthase. By manipulating key enzymes in the sterol pathway, such as 3-hydroxy-3-methylglutaryl-CoA reductase and lanosterol synthase, the researchers achieved a 50% increase in β-amyrin production, reaching levels of 6 mg/L in culture.

In a related study, Tessa et al. identified three OSC genes in *A. annua* that play crucial roles in the biosynthesis of specialized triterpenoids for the cuticle of aerial organs [18]. Focusing on the plant’s aerial organs, the authors identified two key enzymes, OSC2 and CYP716A14v2, through comparative transcriptome analysis of glandular and filamentous trichomes. OSC2, a multifunctional oxidosqualene cyclase, catalyzes the cyclization of 2,3-oxidosqualene into α-amyrin, β-amyrin, and δ-amyrin, with a preference for α-amyrin. CYP716A14v2, a cytochrome P450 oxidase, oxidizes the C-3 hydroxyl group of these triterpenes to ketones, yielding α-amyrone and β-amyrone. The other two OSC genes encoded cycloartenol synthase and lupeol synthase, respectively. This finding underscores the functional diversity within the OSC gene family. The identified OSC genes encode enzymes that produce triterpenoids with unique structures and functions, contributing to the formation of the wax layer on the cuticle of *A. annua*. This wax layer likely serves as a protective barrier against biotic and abiotic stress.

While these studies have provided valuable insights into the biochemical functions of individual OSC genes, a comprehensive genome-wide analysis of the OSC gene family in A. annua is still lacking. Such an analysis would enable a more thorough understanding of the genetic basis for triterpene biosynthesis in this medicinal plant, potentially leading to the discovery of novel OSC genes and the development of related biotechnological applications.

In this study, we aim to address this knowledge gap by conducting a genome-wide identification and characterization of the OSC gene family in *A. annua*. We will employ bioinformatics tools to identify all putative OSC genes in the *A. annua* genome and analyze their phylogenetic relationships, gene structures, conserved domain, motifs, amino acid sequences, and protein 3D structures. Furthermore, we will investigate the expression patterns of these OSC genes across different tissues of *A. annua* using publicly available transcriptome data. This comprehensive analysis will offer valuable insights into the evolution and functional diversification of the OSC gene family in *A. annua*. It will lay the foundation for future studies focused on manipulating triterpenoid biosynthesis in this plant.

## 2. Materials and Methods

### 2.1. Genome Data Acquisition and OSC Gene Identification

The genome data and general feature format (GFF) of *Artemisia annua* were retrieved from the NCBI genome database (https://ftp.ncbi.nlm.nih.gov/genomes/all/GCA/003/112/345/GCA_003112345.1_ASM311234v1/, accessed on 26 April 2025). The OSC protein sequences of *Arabidopsis thaliana* and *Oryza sativa* were obtained from the TAIR database vTAIR10 (https://www.arabidopsis.org/, accessed on 26 April 2025) and the Rice Genome Annotation Project database vMSU7.0 (https://rice.plantbiology.msu.edu/, accessed on 26 April 2025), respectively. Hidden Markov model (HMM) profiles for PF13243 and PF13249 were downloaded from the InterPro database v95.0 (https://www.ebi.ac.uk/interpro/, accessed on 26 April 2025). Subsequently, the HMMER 3.0 software was employed to identify AaOSC proteins in *Artemisia annua* with an *E*-value threshold of ≤1 × 10^−5^ and a sequence similarity exceeding 50%. All other parameters used the default settings. Additionally, the BLASTP algorithm was utilized to search for homologous protein sequences in *Artemisia annua*, using the OSC protein sequences of *Arabidopsis thaliana* and rice as queries, under an same *E*-value ≤ 1 × 10^−5^ and sequence similarity > 50%. All other parameters used the default settings. All candidate AaOSC protein sequences were further analyzed to confirm the presence of the OSC domain. Finally, the longest transcript for each gene was selected using the R package seqfinder v1.2.3 (https://github.com/yueliu1115/seqfinder, accessed on 28 April 2025).

### 2.2. Physicochemical Properties Analysis of AaOSCs

The protein sequences of OSC genes identified in *Artemisia annua*, *Arabidopsis thaliana*, *Helianthus annuus*, *Oryza sativa* Japonica, and *Vitis vinifera* were retrieved from genomic annotation data. Key physicochemical properties were calculated for each OSC protein sequence using the Peptides package (version 2.4.5) in R. The calculated properties included: protein length (number of amino acid residues), molecular weight (MW), hydrophobicity (quantified as the Grand Average of Hydropathy, GRAVY index), and isoelectric point (pI). The resulting data were statistically analyzed and visualized using ggplot2 v3.4.4.

### 2.3. Gene Structure, Protein Feature Analysis, and Phylogenetic Analysis

The domain architectures of gene family members were predicted using Pfam-scan v1.6 (*E*-value cutoff 0.001) against the Pfam 35.0. For de novo motif discovery, the MEME Suite v5.5.2 (https://meme-suite.org/meme/index.html, accessed on 28 April 2025) was utilized in Classical mode with a maximum of 10 motifs and motif widths between 10 and 100 nucleotides. Gene structural features (exon–intron organization) were retrieved from the species’ genome annotation file in GFF3 format. Multiple sequence alignment was conducted using SeaView v5.1 with the MUSCLE algorithm under default parameters. All nucleotide sequences were manually trimmed and edited. Phylogenetic reconstruction was performed in MEGA-X (v11.0.13) using the neighbor-joining method with the Jones–Taylor–Thornton (JTT) substitution model, uniform rates, and 1000 bootstrap replicates. The visualization of the phylogenetic tree was performed using the FigTree v1.4.4.

### 2.4. Promoter Cis-Element and Functional Annotation

The 2000 bp promoter sequences of AaOSC family genes were extracted from the genomic files (GFF). The website of PlantCARE (https://bioinformatics.psb.ugent.be/webtools/plantcare/html/, accessed on 29 April 2025) was utilized to conduct a statistical analysis of cis-element prediction for each gene promoter. Gene Ontology (GO) functional annotation of the plant species examined in this study was performed with eggNOG-mapper v2.1.9 (default parameters) across Biological Process, Cellular Component, and Molecular Function ontologies [38]. Subsequent statistical analyses and data visualization were carried out in R. To explore the regulatory network of AaOSC protein, we used the AraNet2 database (weight > 4) to predict its interaction network.

### 2.5. Expression Profiling

To explore the expression pattern of the AaOSC gene during photomorphogenesis, RNA-seq datasets of *Artemisia annua* exposed to UV-B radiation were retrieved from the NCBI SRA database (accession PRJNA669851). *A. annua* seeds were sown in pots filled with a 3:7 mixture of peat soil and vermiculite and placed in a greenhouse under the growth conditions of 16 h light (Osram 16w-865-AC; 3000 lx)/8 h dark photoperiod at 23 ± 2 °C, supplemented with regular water and fertilizer. Six-week-old seedlings were exposed daily to narrow-band UV-B radiation (Philips TL20W/01RS; 1.5 μmol·m^−2^·s^−1^) for 4 h (10:00–14:00) over 9 days under standard growth conditions. The youngest fully expanded leaf was collected at 0, 2, 4, and 6 h post-treatment, immediately flash-frozen in liquid nitrogen, and stored at −80 °C until RNA extraction for downstream expression analysis. RNA-seq reads were aligned to the *A. annua* CTI1.2 genome using HISAT2 v2.2.0 (default parameters). Gene-level quantification was performed with featureCounts v1.6.4 (union mode; multi-mapped reads excluded), followed by TPM normalization. Differentially expressed genes were visualized via hierarchical clustering using Pheatmap v1.0.12 (distance metric: Euclidean; linkage method: complete). Statistical significance (*p* < 0.05) was determined by ANOVA with Tukey’s HSD post hoc testing.

## 3. Results

### 3.1. Genome-Wide Identification of AaOSCs and Physical and Chemical Characteristics

**Figure 1:**
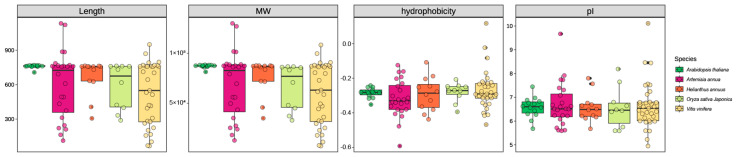
Comparative analysis of physicochemical properties of oxidosqualene cyclase (OSC) genes across five plant species. Gene length distribution (amino acid count). Molecular weight (MW, kDa) of OSC proteins. Hydrophobicity (GRAVY index). Isoelectric point (pI). Species: *Arabidopsis thaliana*, *Artemisia annua*, *Helianthus annuus*, *Oryza sativa Japonica*, and *Vitis vinifera*.

The physicochemical properties of the oxidosqualene cyclase (OSC) gene family were analyzed across five plant species: *Artemisia annua*, *Arabidopsis thaliana*, *Helianthus annuus*, *Oryza sativa Japonica*, and *Vitis vinifera*. The analysis encompassed gene length, molecular weight (MW), hydrophobicity, and isoelectric point (pI) (Figure 1, Data S1 and S2). The distribution of gene lengths varied among the species. *Artemisia annua* exhibited the largest range in gene length, from approximately 300 to nearly 1000 amino acids. *Arabidopsis thaliana* showed a relatively constrained distribution, clustered around 700 amino acids. *Helianthus annuus*, *Oryza sativa Japonica*, and *Vitis vinifera* displayed intermediate ranges and median values. Similarly to gene length, the MW distribution was broadest in *Artemisia annua*, ranging from approximately 30 kDa to >100 kDa. The remaining species exhibited narrower MW distributions, with medians between approximately 60 kDa and 80 kDa. The predicted hydrophobicity of the OSC proteins was generally negative across all species, indicating a predominantly hydrophobic nature. *Vitis vinifera* displayed the widest range of hydrophobicity values, extending to approximately 0.0, whereas *Arabidopsis thaliana* showed the narrowest and most negative range. The predicted pI values of the OSC proteins ranged from approximately 5.5 to 8.5. *Artemisia annua* showed the widest distribution of pI values, with some members approaching a pI of 9. *Vitis vinifera* exhibited a subset of members with pI values exceeding 8. The other species exhibited more constrained pI ranges centered around a pI of 6.5.

### 3.2. Gene Structure, Conserved Domain and Motif Analysis

**Figure 2:**
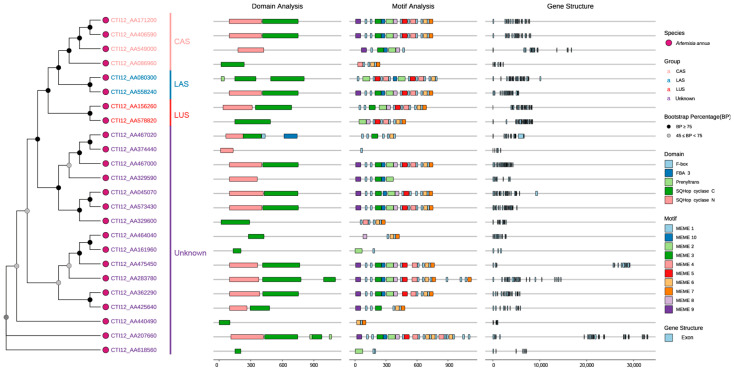
Phylogenetic relationships, domain architecture, conserved motifs, and gene structure of AaOSC genes.

Phylogenetic analysis of 24 AaOSC genes resolved four functional clades: CAS (cycloartenol synthase), LAS (lanosterol synthase), LUS (lupeol synthase), and an evolutionarily divergent unknown clade, with most nodes showing strong bootstrap support (≥75%) and partial support (45–75%) for minor branches (Figure 2). Subsequent domain architecture analysis confirmed canonical OSC functions through conserved SQHop cyclase N-terminal and C-terminal domains across all members, though CTI12_AA467020 (unknown clade) uniquely encoded a C-terminal F-box domain, suggesting potential post-translational regulatory mechanisms distinct from typical OSCs. Furthermore, clade-specific motif organization revealed core signatures in the CAS clade despite significant variations in motif order/composition within clades (e.g., CTI12_AA425640 vs. CTI12_AA2077660). This variability was mirrored in gene structure complexity, where exon numbers ranged widely (12–26) and intron lengths exhibited pronounced divergence, particularly in CTI12_AA475450 and CTI12_AA207660, consistent with functional diversification across the gene family.

### 3.3. Promoter Cis-Elements

**Figure 3:**
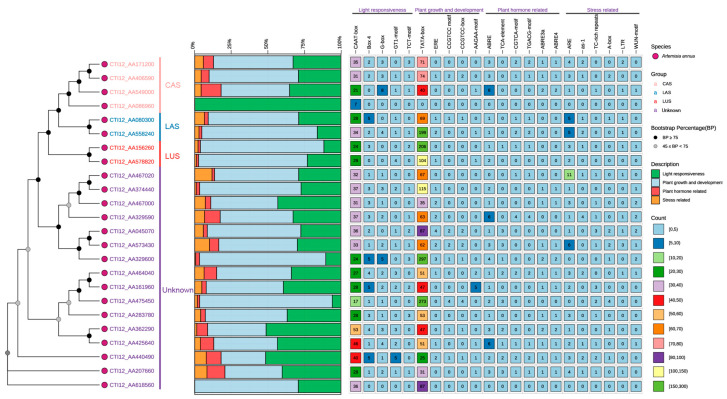
Cis-regulatory element analysis of AaOSC gene promoters. The phylogenetic tree on the left indicates the relationships between the 24 AaOSC genes analyzed. The stacked bar chart displays the relative proportion of CRE categories (light responsiveness, plant growth and development, plant hormone-related, and stress-related). The heatmap represents the number of each CRE type found in the promoter region of each gene. The color intensity in the heatmap corresponds to the number of elements as indicated in the legend.

Analysis of cis-regulatory elements (CREs) in promoters of 24 AaOSC genes revealed clade-specific regulatory signatures across four functional categories: light responsiveness, growth/development, hormone signaling, and stress response (Figure 3). Specifically, the CAS clade was dominated by light-responsive elements (e.g., G-box), while the LUS clade showed enrichment for growth/development motifs. Conversely, LAS members exhibited pronounced enrichment of stress-response elements, and the unknown clade displayed exceptional CRE diversity. Notably, at the gene level, CTI12_AA467020 (unknown clade) contained the highest density of antioxidant response elements (ARE), whereas CTI12_AA549000 (CAS) showed maximal ABA-responsive elements (ABRE). Additionally, LAS genes CTI12_AA080300 and CTI12_558240 harbored significant ARE clusters. Collectively, key stress-related CREs, including ARE, as-1, TC-rich repeats, LTR, and WUN-motif, demonstrated adaptive regulatory specialization within the OSC family.

### 3.4. Phylogenetic Analysis

**Figure 4:**
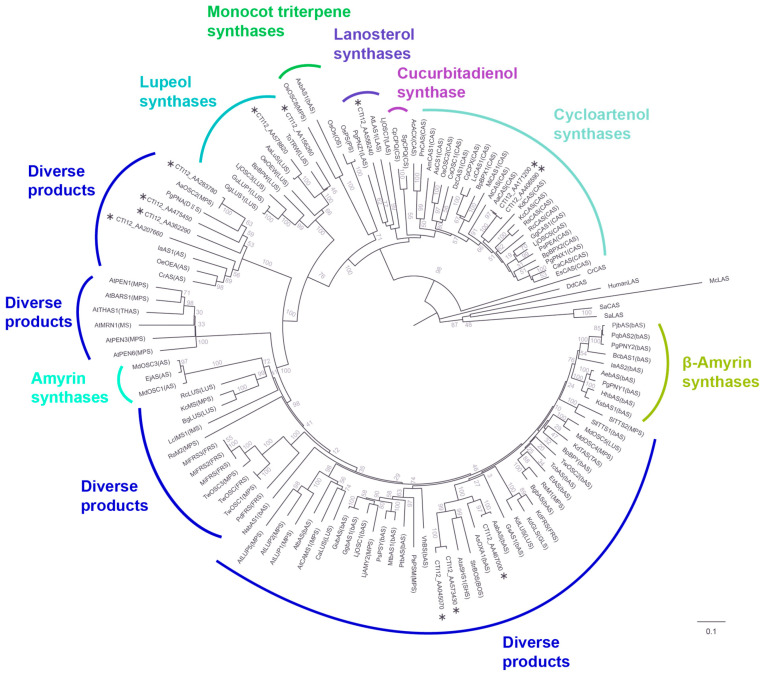
Phylogenetic relationships of AaOSC genes. The tree was constructed using neighbor-joining method based on the amino acid sequences of 12 AaOSC genes and other OSCs from various plant species. AaOSC genes are marked with asterisks. Numbers at the nodes indicate bootstrap support values (%).

A phylogenetic tree was constructed to investigate evolutionary relationships and other functionally characterized OSCs from diverse plant species (Figure 4, Table S1). In the phylogenetic reconstruction, putative pseudogenes encoding shorter proteins were excluded through stringent quality control, with 12 members ultimately incorporated into the final phylogenetic tree. The analysis revealed distinct clades corresponding to known OSC functionalities, as well as subclades containing AaOSC genes. The tree exhibited clear separation of OSCs based on their known enzymatic activity. The AaOSC genes CTI12_AA578820 and CTI12_AA156260 cluster within the lupeol synthase clade. These AaOSCs exhibited strong bootstrap support for their placement within this clade, indicating high confidence in their relatedness to known lupeol synthases. CTI12_AA171200 and CTI12_AA406590 are grouped within the cycloartenol synthase clade, sharing a close evolutionary relationship with cycloartenol synthases from other plant species. CTI12_AA558240 falls within the lanosterol synthase clade, which is known to produce lanosterol, a precursor to steroidal compounds. CTI12_AA283780, CTI12_AA475450, CTI12_AA362290, CTI12_AA207660, CTI12_AA045070, CTI12_AA573430, and CTI12_AA467000 are positioned among clades producing diverse products without clustering strongly with any functionally defined groups, suggesting the potential for neofunctionalization.

### 3.5. GO Annotation

**Figure 5:**
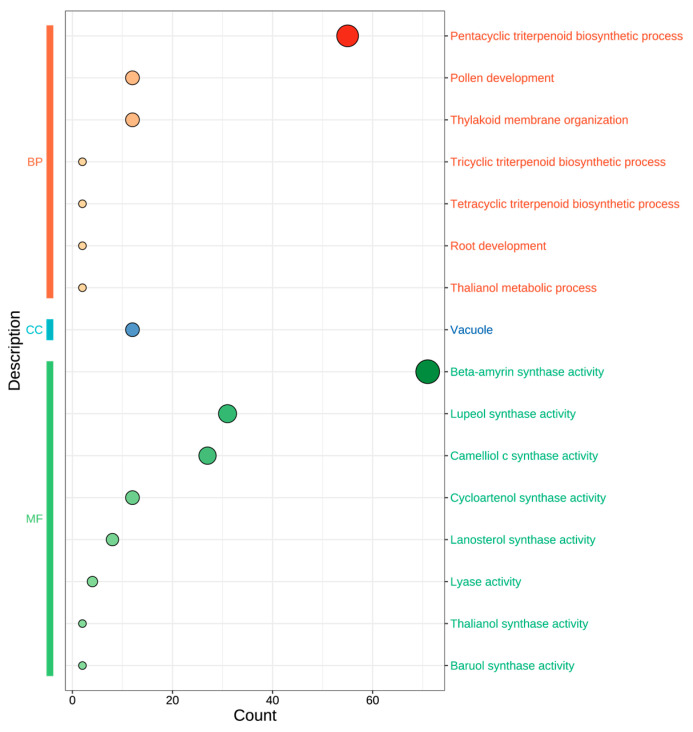
Gene Ontology (GO) enrichment analysis of AaOSC genes. GO terms are categorized into Biological Process (BP), Cellular Component (CC), and Molecular Function (MF). The size of each circle represents the number of AaOSC genes associated with the corresponding GO term, and the *x*-axis indicates the number of genes associated with each GO term.

To elucidate the functional implications of the 24 AaOSC genes, Gene Ontology (GO) enrichment analysis was performed (Figure 5). Enriched GO terms were categorized into three main ontologies: Biological Process (BP), Cellular Component (CC), and Molecular Function (MF). As illustrated in Figure 5, the size of the circles corresponds to the number of genes associated with each GO term, indicating the relative enrichment. Within the biological process category, “pentacyclic triterpenoid biosynthetic process” showed the highest enrichment. Other significantly enriched biological processes included “pollen development” and “thylakoid membrane organization.” The most enriched cellular component term was “vacuole.” In the molecular function category, several OSC activities were enriched, with “beta-amyrin synthase activity” being the most prominent. Other notable molecular functions identified included “lupeol synthase activity”, “camelliol c synthase activity”, “cycloartenol synthase activity”, “lanosterol synthase activity”, “lyase activity”, “thalianol synthase activity”, and “baruol synthase activity”.

### 3.6. Protein–Protein Interaction

**Figure 6:**
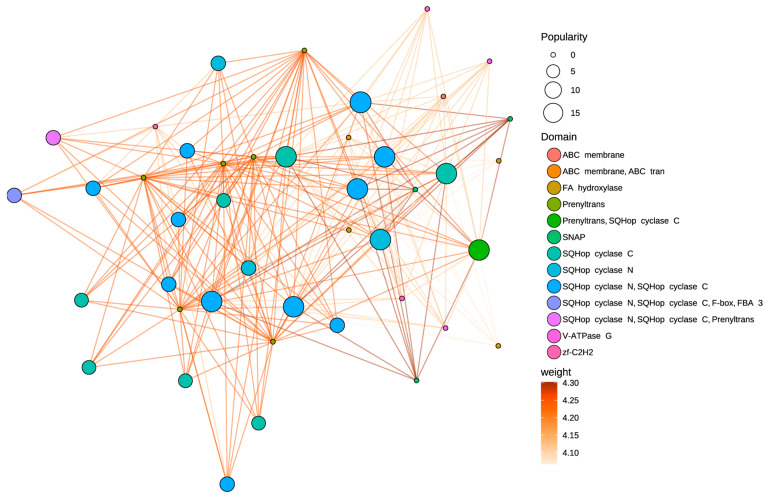
Predicted protein–protein interaction (PPI) network of AaOSC genes. Nodes represent proteins, and edges represent predicted interactions. Node size indicates ‘popularity’ (number of interactions). Node color indicates the protein domain. Edge color indicates the interaction weight based on a gradient from light orange (lower weight) to dark orange (higher weight).

The PPI network displays significant interconnectivity among AaOSC family members (Figure 6). Proteins containing SQHop cyclase domains (specifically SQHop cyclase N, SQHop cyclase C, and combinations) constitute a major interacting group within the network. Nodes representing proteins with both SQHop cyclase N and SQHop cyclase C domains display consistently high popularity. OSC proteins annotated with both SQHop cyclase N and SQHop cyclase C domains are observed with interaction weights ranging from 4.25 to 4.30, connecting them to proteins with similar domain architectures. A distinct subnetwork is formed by proteins containing the Prenyltrans domain. This subnetwork is closely associated with SQHop cyclase domain-containing proteins. One notable observation is the strong interaction (weight = 4.30) between a protein containing both Prenyltrans and SQHop cyclase C domains and proteins containing only the Prenyltrans domain. The ABC membrane domain-containing proteins show peripheral locations in the network, linked to the SQHop cyclase cluster through lower-weight interactions.

### 3.7. Expression Pattern in Tissues and Under UV Pressure

**Figure 7:**
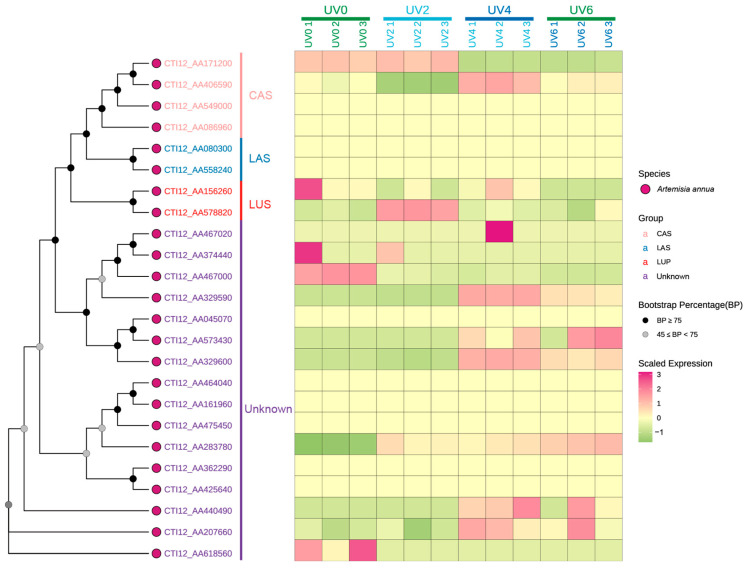
Heatmap representation of the expression profiles of AaOSC genes under UV irradiation. Columns correspond to different UV treatment conditions (0 h, 2 h, 4 h, and 6 h of UV exposure), with numbers indicating replicates. Bootstrap values for the phylogenetic tree are indicated by node shading.

The expression patterns of AaOSC genes were analyzed under various UV irradiation treatments (0 h, 2 h, 4 h, and 6 h of UV exposure) using heatmap visualization (Figure 7, Table S2). A phylogenetic tree showing the relationships among the analyzed AaOSC genes is displayed alongside the heatmap. The heatmap reveals distinct expression patterns across the different AaOSC gene family members and UV treatments. Most of the genes show differences in gene expression between the different UV exposure levels. Genes belonging to the CAS and LAS subfamilies generally exhibited relatively low expression levels across all UV treatments compared to the other groups. Notably, CTI12_AA406590 exhibited relatively higher expression in the UV4 condition compared to other CAS family members. The expression of the LUS subfamily showed variation under different UV treatments. CTI12_AA156260 displayed relatively high expression in the UV0 condition, while CTI12_AA578820 appeared to be upregulated in the UV2 condition. Genes from the unknown subfamily showed distinct expression patterns. Notably, CTI12_AA467020 was upregulated in the UV4 conditions. Several genes within the unknown subfamily showed minimal changes in expression. However, CTI12_AA440490 expression notably increased in UV4.

## 4. Discussion

### 4.1. Oxidosqualene Cyclase (OSC) Functional Diversification in A. annua

Oxidosqualene cyclase (OSC) is a key enzyme in plant secondary metabolism that controls triterpene and sterol biosynthesis. As a membrane-associated enzyme, OSC catalyzes the stereospecific cyclization of 2,3-oxidosqualene into triterpenoid scaffolds (e.g., α-amyrin, β-amyrin, lupeol, taraxerol, friedelin) or sterol precursors (e.g., cycloartenol, lanosterol), depending on its isoform specific activity [3,13,39]. Critically, OSC function is constrained by physicochemical properties that dictate subcellular localization, stability, and interactions with membrane-bound partners. Our comparative analysis of OSC proteins across five angiosperms (*A. annua*, *A. thaliana*, *H. annuus*, *O. sativa*, *V. vinifera*) revealed that AaOSCs exhibit exceptional structural plasticity (Figure 1). This cross-species comparison across Asteraceae, Brassicaceae, Poaceae, and Vitaceae evaluates whether extreme physicochemical traits are linked to ecological specialization. *A. annua*’s expanded OSC repertoire displays unparalleled variability in gene length, MW, and pI, suggesting evolutionary innovations to accommodate diverse substrates or regulatory mechanisms. This regulates metabolic flux toward defense-related triterpenoids (e.g., saponins) or essential phytosterols (e.g., sitosterol, stigmasterol), balancing plant growth and environmental adaptation [13].

Our genome-wide analysis identified 24 OSC genes (AaOSCs) in *Artemisia annua*, revealing a metabolic toolkit more expansive than model species like *Arabidopsis thaliana* [15,16,40]. Phylogenetic analysis of a robust subset of 12 putatively functional AaOSCs revealed that the majority (7/12) clustered within clades associated with diverse or unspecified products. This intriguing distribution, alongside the expansion in specialized clades (e.g., cycloartenol, lanosterol, lupeol), highlights the potential for catalytic promiscuity or neofunctionalization within the AaOSC family. Such plasticity could be a key driver enabling *A. annua*’s diverse triterpenoid profile and ecological adaptability, including its production of pharmacologically active compounds like artemisinin precursors [41]. Notably, the LUS clade encompasses evolutionarily conserved genes that encode enzymes responsible for synthesizing lupeol and its derivatives [13,32]. These bioactive triterpenoids serve critical defensive roles in plants, such as antimicrobial protection, ROS scavenging, and herbivore deterrence [11,12,13]. The multifunctionality of lupeol derivatives highlights their ecological significance in plant stress adaptation. These findings underscore the evolutionary diversification of OSCs in *A. annua*. This diversification may have been driven by selective pressures to optimize defense metabolites while maintaining sterol homeostasis [12,13].

### 4.2. Evolutionary Mechanisms Enabling Metabolic Innovation

The phylogenetic segregation of AaOSCs into CAS, LAS, LUS, and unknown subfamilies mirrors catalytic specialization observed in other plants but reveals unique features. The atypical occurrence of LAS-like genes in *A. annua* raises questions about potential horizontal gene transfer (HGT) from non-plant lineages, a mechanism documented in terpene synthase evolution [3]. This HGT event may have conferred adaptive advantages under abiotic stress, as lanosterol-derived sterols enhance membrane stability in fluctuating environments [42,43,44]. This is a hypothesis supported by the enrichment of abiotic stress-responsive cis-elements in CTI12_AA080300 and CTI12_AA558240 promoters. This evolutionary plasticity aligns with the “gene toolkit” model, where HGT expands metabolic repertoires in sessile organisms facing environmental pressures [15,40,43]. However, definitive evidence for HGT requires expanded phylogenomic analysis beyond the scope of this study. Future work will incorporate fungal and animal OSC sequences to test this hypothesis. Experimental validation of CTI12_AA558240’s activity could redefine our understanding of sterol pathway plasticity in plants. Additionally, exon–intron variability among AaOSCs suggests regulatory divergence, such as tissue-specific splicing, which may fine-tune triterpenoid production under stress [41,45,46]. The discovery of an F-box domain in CTI12_AA467020 suggests a candidate mechanism for post-translational regulation via ubiquitination, a mechanism known to modulate secondary metabolism in response to environmental cues [47,48,49]. However, this model requires direct experimental validation to confirm ubiquitin ligase activity and substrate specificity The structural plasticity of unknown clade members, positioned diverse products clades, highlights a “transitional zone” for catalytic promiscuity [40,50]. Minor active-site variations, including residue substitutions in the QW motif, DCTAE loop, or other regions, could enable product switching, a mechanism proposed to drive metabolic diversity in *Arabidopsis*, *Avena strigose* and rice [3,40,51,52,53]. This plasticity may explain the expansion of triterpenoid profiles in *A. annua*, a species renowned for its ecological resilience [40,41]. However, these predictions require biochemical validation (e.g., heterologous expression assays) to definitively establish enzymatic activities. Future research must prioritize biochemical characterization of unknown-clade AaOSCs. For example, heterologous expression in yeast or transient plant systems coupled with metabolic profiling (GC-MS, LC-MS/MS) can resolve their catalytic products and test plasticity hypotheses.

### 4.3. Transcriptional Regulation as an Adaptive Interface

Promoter analysis uncovered clade-specific cis-elements, linking transcriptional regulation to physiological roles. Promoter cis-element analysis revealed that AaOSCs are tightly regulated by hormonal and environmental cues. These findings echo studies in *Glycyrrhiza uralensis*, where ABA and JA signaling co-regulate triterpenoid biosynthesis under stress [17]. The enrichment of ABA/JA-responsive motifs (TGACG/CGTCA motif) in LUS promoters aligns with lupeol’s role in biotic stress responses [54], while light-responsive elements in CAS promoters may synchronize sterol biosynthesis with photosynthetic activity [42,55]. Strikingly, the UV-B-responsive HY5-binding sites (G-box) prevalent across AaOSCs suggest co-regulation with photoprotective pathways, akin to UV-induced flavonoid biosynthesis in grapevines [56,57]. These regulatory signatures, combined with the abiotic stress motifs in LAS promoters (e.g., MYC, LTR), position AaOSCs at the nexus of environmental adaptation, enabling *A. annua* to dynamically allocate resources between growth and defense.

### 4.4. Expression Dynamics Reflect Functional Prioritization

The low basal expression of most CAS subfamily members aligns with observations in *Arabidopsis* and *Medicago*, where CAS-like enzymes are predominantly regulated by developmental cues rather than abiotic stress [58,59,60]. The transient upregulation of CTI12_AA171200 and CTI12_AA406590 at UV2 suggests a stress-responsive role distinct from canonical CAS functions, possibly linked to triterpenoid phytoalexin biosynthesis hypothesized in plants [3,59]. The upregulation of LUS subfamily members, specifically CTI12_AA578820 in UV4 aligns with the known role of lupane-type triterpenoids in UV-B shielding and membrane stabilization [14]. This response mirrors the UV-inducible β-amyrin synthase activity in *Glycyrrhiza uralensis* [61], suggesting conserved adaptive strategies across eudicots [11,39]. Notably, the unknown subfamily member CTI12_AA440490 exhibits UV4-specific upregulation. Phylogenetic divergence from characterized OSCs suggests this isoform may catalyze novel triterpenoid scaffolds, analogous to the recently discovered marneral synthase in *A. thaliana* [62,63]. Such cryptic metabolic pathways could represent untapped reservoirs for bioactive compound discovery, particularly in stress-adapted species like *A. annua* [64].

### 4.5. Bridging Knowledge Gaps

While our study provides a genomic foundation, key gaps remain. First, functional characterization of unknown AaOSCs is critical. Heterologous expression in yeast or N. benthamiana could elucidate products. However, in planta assays may better capture substrate availability and regulatory dynamics [65,66,67,68,69]. Second, cis-element predictions do not account for epigenetic modifications or transcription factor availability; chromatin immunoprecipitation (ChIP-seq) or CRISPR-edited promoter studies could validate motif functionality [70,71,72]. Third, expression profiling under diverse stresses (e.g., herbivory, drought) and tissues (e.g., glandular trichomes, roots) is needed to resolve spatiotemporal roles, particularly for clades with weak phylogenetic clustering [15,73,74]. Finally, protein structure prediction of AaOSCs (e.g., via AlphaFold2) could reveal catalytic residues and substrate-binding pockets, enabling rational engineering to enhance triterpenoid yields [75,76].

### 4.6. An Engineering Roadmap

The AaOSC repertoire offers a roadmap for metabolic engineering. Modulating CAS/LAS expression (e.g., via CRISPR-Cas9) may theoretically redirect FPP flux toward artemisinin precursors, given shared MVA pathway dependence. However, this model requires validation of substrate competition dynamics. Furthermore, resolving the evolutionary origin of LAS-like enzymes through comparative genomics could uncover HGT mechanisms applicable to crop improvement. Beyond *A. annua*, this study underscores the value of integrating phylogenomics, regulatory element analysis, and structural modeling to unravel metabolic diversity in medicinal plants.

## 5. Conclusions

This study provides a comprehensive genome-wide analysis of the OSC gene family in *Artemisia annua*, revealing a repertoire of 24 AaOSC genes with diverse phylogenetic relationships, structural features, and expression patterns. The classification of these genes into CAS, LAS, LUS, and unknown subfamilies suggests functional specialization in sterol and triterpenoid biosynthesis. The identification of cis-regulatory elements in AaOSC promoters highlights their responsiveness to light, hormones, and stress signals, indicating a critical role in environmental adaptation. Expression analysis under UV-B irradiation revealed differential regulation of AaOSC genes, further supporting their involvement in stress responses. These genomic resources directly enable biotechnological applications: CRISPR-Cas9 editing of LAS genes may enhance artemisinin yield by redirecting metabolic flux, while UV-responsive promoters (e.g., CTI12_AA467020) offer synthetic biology tools for stress-inducible triterpenoid production. The presence of LAS-like enzymes raises the possibility of horizontal gene transfer and highlights evolutionary plasticity in this medicinal plant. The AaOSC repertoire offers a roadmap for metabolic engineering strategies aimed at enhancing triterpenoid production and stress tolerance in *A. annua*. Future research should focus on functional characterization of the unknown AaOSCs, validation of cis-element functionality, and expression profiling under diverse stress conditions to fully elucidate the spatiotemporal roles of these genes.

## Data Availability

The data and materials that support the findings of this study were available from the corresponding author upon reasonable request.

## Supplementary Materials

The following supporting information can be downloaded at: https://www.mdpi.com/article/10.3390/cimb47070545/s1.
